# Inflammatory biomarkers in workers exposed to silica dust: integrative review

**DOI:** 10.47626/1679-4435-2023-1224

**Published:** 2024-11-14

**Authors:** Patricia Canto Ribeiro, Tatiana Paula Teixeira Ferreira, Marco Aurélio Martins, Patrícia Machado Rodrigues e Silva Martins, Hermano Albuquerque de Castro

**Affiliations:** 1 Escola Nacional de Saúde Pública Sérgio Arouca, Fundação Oswaldo Cruz (Fiocruz), Rio de Janeiro, RJ, Brazil; 2 Laboratório de Inflamação do Instituto Oswaldo Cruz, Fiocruz, Rio de Janeiro, RJ, Brazil

**Keywords:** free silica, silicosis, biomarkers, cytokines, chemokines, sílica livre, silicose, biomarcadores, citocinas, quimiocinas

## Abstract

**Introduction:**

Silicosis is a severe, progressive, fibrosing lung disease caused by the inhalation of free crystalline silica dust; it is the most prevalent pneumoconiosis worldwide. It is associated with a chronic inflammatory process triggered by silica particles in the pulmonary alveoli. Alveolar macrophages play a key role in the pathogenesis of silicosis, with additional contributions from polymorphonuclear cells, epithelial cells, and the release of inflammatory mediators.

**Objectives:**

To compile updated information on key inflammatory biomarkers in workers exposed to silica.

**Methods:**

Integrative review to discuss the state of the art regarding major biomarkers used in the early diagnosis and search for treatments for workers exposed to silica. The SciELO and PubMed databases were searched for articles published from 2012 to 2022.

**Results:**

The search strategy retrieved 111 articles, of which 29 were duplicates across the two databases. Of the 82 remaining articles, 67 were excluded after screening of abstracts (review articles, articles on polymorphisms/genetics, and animal studies). Fifteen articles were read in full; of these, two were eliminated as they did not meet the inclusion criteria. Of the 13 articles retained for analysis, 12 were cross-sectional and only 1 was a prospective observational study.

**Conclusions:**

This integrative review identified the importance of cytokines in silica-related illness. This can help encourage future research and guide the development of new therapies and interventions for silicosis.

## Abstract (pt)

**Introdução:**

A silicose é uma doença pulmonar grave, progressiva e fibrosante, causada pela inalação de poeira de sílica cristalina livre, sendo a pneumoconiose de maior prevalência no mundo. Associada a um processo inflamatório crônico desencadeado pela partícula de sílica no alvéolo pulmonar, tem como centro do processo o papel do macrófago alveolar, o aporte de polimorfonucleares, a participação das células epiteliais e a liberação de mediadores inflamatórios.

**Objetivos:**

Atualizar as informações sobre os principais biomarcadores inflamatórios em trabalhadores expostos à sílica.

**Métodos:**

Revisão integrativa para discutir o estado da arte para os principais biomarcadores utilizados para diagnóstico precoce e busca de tratamentos para trabalhadores expostos à sílica. Foram realizadas buscas por palavras-chave nas bases de dados SciELO e PubMed, no período de 2012 a 2022.

**Resultados:**

Foram encontrados 111 artigos, sendo 29 duplicados nas duas bases. Dos 82 artigos restantes, foi realizada a leitura dos resumos, e foram excluídos 67 artigos por se tratarem de artigos de revisão, artigos sobre polimorfismos, genética e estudos em animais. Quinze artigos foram lidos na íntegra, e, após a leitura, mais dois artigos foram eliminados por não atenderem aos critérios de inclusão. Seguiu-se, então, à análise de 13 artigos, 12 transversais e apenas 1 estudo observacional prospectivo.

**Conclusões:**

Foi possível identificar a importância das citocinas no adoecimento pela sílica. Isso pode contribuir para incentivar futuras pesquisas e orientar o desenvolvimento de novas terapias e intervenções para a doença.

## INTRODUCTION

Silicosis is the most prevalent occupational disease worldwide. It is a severe, progressive, fibrosing lung disease caused by inhalation of respirable crystalline silica (silica dust).^1^ Its manifestations are not limited to physical illness, but also extend to psychological suffering. Few therapeutic resources are available for those affected, whether curative (lung transplantation), palliative (with measures that can prolong survival, such as bronchoalveolar lavage),^2^ or supportive. This makes the search for disease-modifying treatments capable of slowing the progression of silicosis a pressing unmet need for thousands of workers across the world.

Factors associated with the development of silica-associated illness include individual susceptibility, exposure time, and quantity and characteristics of the inhaled dust.^3^ Once the inhaled particles reach the pulmonary alveoli (the gas exchange zone), they undergo phagocytosis by alveolar macrophages, triggering and perpetuating a cyclical inflammatory process which has yet to be fully elucidated.^4^

Host defense cells-macrophages and neutrophils-are implicated in the inflammatory process. As macrophages are normally found in the alveolar space, they are the first resident cells to make contact with silica.^5^ They are present in large numbers in the pulmonary interstitium and within the alveoli, thus constituting a significant and effective part of the innate immune response. Macrophages also play a key role in the tissue repair process, as they have the ability to secrete cytokines and chemotactic factors that regulate the local accumulation of mesenchymal cells (fibroblasts) and extracellular matrix components.

Despite extensive research into inflammation and fibrosis, little is known about the core cellular mechanisms that initiate and direct the inflammatory process. According to recent work by Martins et al.,^6^ the protein Found in Inflammatory Zone 1 (FIZZ1), produced by alveolar macrophages and fibroblasts, is capable of inducing the proliferation and transdifferentiation of myofibroblasts, causing tissue fibrosis; this reaffirms the role of macrophages and fibroblasts in the pathogenicity of silica-associated illness. Martins et al.^6^ further suggest that autoimmunogenic type V collagen, produced by alveolar epithelial cells and fibroblasts, associated with FIZZ1, Notch-1, and the peroxisome proliferator-activated receptor-γ (PPARγ), may be involved as a key pathogenic mechanism in the formation of silicotic granulomas in the mouse lung. Mice have been used as models to study the pathophysiological factors involved in the pathogenesis of several illnesses and of the inflammatory process in humans.

Interleukin-13 (IL-13) is a key mediator of tissue fibrosis caused by inflammation. Secreted by type 2 (Th2) T helper cells,^7^ is plays a key role in various inflammatory and pathogenic processes, such as granuloma formation, and has been studied for potential associations with several conditions, such as neoplastic diseases and non-alcoholic fatty liver disease.^8^ An anti-silicosis therapeutic strategy specifically aimed at the profibrotic activity of IL-13 in the lung has been studied in mice. It involves a fusion protein (IL-13-PE38QQR or IL-13-PE) composed of a fragment of human IL-13, which recognizes and binds to receptors of this cytokine, and a mutant form of pseudomonal exotoxin.^9^ Preliminary findings suggest this may be a way to reduce the pulmonary inflammation induced by silica dust exposure. Preclinical models demonstrate the key role of inflammation in the pathogenesis of silicosis; Therefore, the identification of inflammatory biomarkers in workers exposed to silica is an interesting approach, as these markers could represent druggable targets for potential disease-modifying treatments.

Four main basic mechanisms of cell toxicity are implicated in silicosis: *a)* direct cytotoxicity of silica, resulting in lung cell damage, release of lipases and proteases, and, eventually, lung fibrosis; *b)* activation of oxidant production by lung phagocytes, which overcomes antioxidant defenses and leads to lipid peroxidation, protein nitrosation, cell injury, and lung fibrosis; *c)* activation of mediator release from alveolar macrophages and epithelial cells, which leads to the recruitment of leukocytes and polymorphonuclear macrophages, resulting in further production of pro-inflammatory cytokines and reactive species and further lung injury and fibrosis; and *d)* secretion of growth factors from alveolar macrophages and epithelial cells, stimulating fibroblast proliferation with subsequent fibrosis.^10^

The inflammatory response is a complex process which involves several types of cells, with a particular focus on white blood cells such as macrophages, neutrophils, and lymphocytes, which release specialized substances including vasoactive amines and peptides, eicosanoids, pro-inflammatory cytokines, and acute phase proteins. These act as mediators of the inflammatory process, preventing further tissue damage and, ultimately, leading to resolution (healing and restoration of tissue function).^11^ Cytokines play important roles in the activity of many cells. Of particular importance is their role in regulating the immune system.^12^ They constitute a class of small proteins that act as signaling molecules at low concentrations (picoor nanomolar) to regulate inflammation and modulate cellular activities, such as growth, survival, and differentiation.^13^ Cytokines are an exceptionally large and diverse class of proor anti-inflammatory factors,^14^ grouped into families based on the structural homology or their receptors.^15^

Chemokines, in turn, are a subgroup of secreted proteins within the cytokine class whose generic function is to induce cell migration.^13^ These “chemotactic cytokines” (hence their name) are involved in leukocyte chemoattraction and trafficking of immune cells to various sites across the body.^13^ Chemokines belong to one of two major categories based on their biological activity: maintaining homeostasis or inducing inflammation. Those that are produced in response to an inflammatory stimulus and facilitate an immune response upon reaching cells of the innate and adaptive immune system are considered pro-inflammatory.^16^ Binding of a cytokine or chemokine to its cognate receptor results in activation, which, in turn, triggers a cascade of signaling events that regulate various cellular functions, such as cell adhesion, phagocytosis, cytokine secretion, cell activation, cell proliferation, cell survival and death, apoptosis, angiogenesis, and proliferation.^11,17^

Several studies have associated cytokines with the inflammatory process which occurs in response to silica exposure, as well as with established silicosis.^18,19^ The present study will focus on some cytokines and chemokines.

Cytokines are proteins that act as mediators of communication between immune system cells and play an important role in the inflammatory response. The most widely studied cytokines are: IL-1α, IL-1β, IL-2, IL-4, IL-6, IL-8, IL-10, IL-12, IL-13, IL-17, IL-18, interferons alpha and gamma (IFN-α and IFN-γ), tumor necrosis factor (TNF)-α, granulocyte colony-stimulating factor (G-CSF), macrophage colony-stimulating factor (M-CSF), granulocyte-macrophage colony-stimulating factor (GM-CSF), transforming growth factor beta (TGF-β), and chemokine ligand 2/monocyte chemoattractant protein-1 (CCL-2/MCP-1). These cytokines play different roles and have targets across the body and are implicated in various diseases and inflammatory processes.

## METHODS

This is an integrative, descriptive, and exploratory literature review, intended to provide an in-depth overview of the topic by evaluating and synthesizing the findings of relevant publications.^20^

Guided by this concept, our research followed the steps suggested by Mendes et al.^20^ The first step consisted of defining the primary objective of the study, while the second defined the inclusion and exclusion criteria. The inclusion criteria were full-text, open-access articles published between 2012 and 2022, containing in their titles the keywords (in English or Portuguese) “cytokines”, “biomarkers”, and “silicosis”. The sole exclusion criterion was duplication of articles across the searched databases. The SciELO and MEDLINE (via PubMed) databases were searched, using different combinations of the aforementioned keywords composed with the aid of the Boolean operators OR/AND.

**Figure 1:**
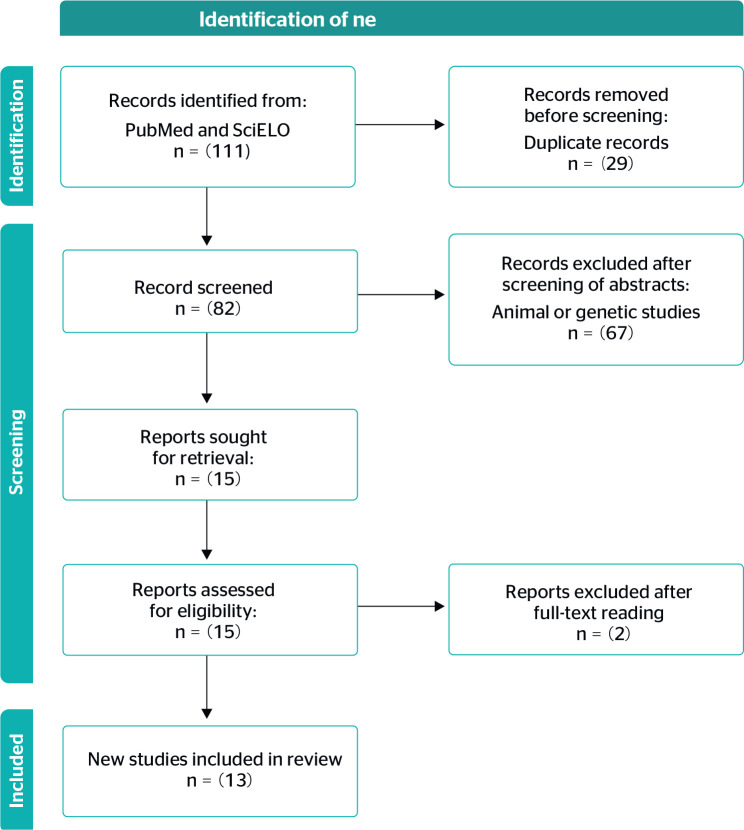
Preferred Reporting Items for Systematic Reviews and Meta-Analyses (PRISMA) flow diagram of study selection.

Once the steps described above had been followed and the search completed, retrieved publications were selected by application of the Preferred Reporting Items for Systematic Reviews and Meta-Analyses (PRISMA) 2020 checklist, which is composed of 27 items. It also includes a flow diagram, which describes the steps of a systematic review.^21^Figure 1 shows the PRISMA flow diagram for the present review.

We conducted an integrative review of articles on the role of cytokines in the pathogenesis of silicosis, published from 2012 to 2022, seeking to describe and discuss the current state of the art regarding use of inflammatory biomarkers of the cytokine class in the early diagnosis and assessment of severity of this condition in workers exposed to silica dust. For this review, only studies in humans which evaluated any biomarker of the cytokine class were included; review articles were not included, nor were articles that evaluated genetic changes.

The search strategy employed the terms “biomarkers” or “cytokines” and “silicosis” in English for the PubMed database and in Portuguese, English, and Spanish for the SciELO database. All searches covered the period between January 2012 and June 2022. A total of 111 articles were retrieved, with 29 duplicates (articles found in both databases). The abstracts of the remaining 82 articles were screened, and a further 67 were excluded because they were review articles, articles on polymorphisms/genetics, and animal studies. Fifteen articles were read in full; after reading, two of these were found not to meet the inclusion criteria and were thus excluded. Thus, 13 articles were ultimately retained for analysis.

## RESULTS

The included studies analyzed a wide range of inflammatory biomarkers in silica-exposed workers, with or without silicosis. Of the 13 articles retained for analysis, 12 were cross-sectional and only 1 was a prospective observational study, conducted in Spain (Table 1).

**Table 1:** Articles selected for the integrative review on inflammatory biomarkers in silica exposure

Authors	Objective(s)	Type of study	Population	Biomarkers	Country
Blanco-Pérez et al.^22^	To determine the clinical significance of specific biomarkers, to estimate their association with the development, severity, and/or progression of silicosis, and identify determinants of this evolution	Prospective observational study from 2009 to 2018	337 exposed to silica (278 with silicosis) and 30 subjects in the control group	IL-6, IL-2R, IL-1B, IL-8, TNF-α, TGF-β1; AAT; CRP; LDH; ferritin	Spain
Scalia Carneiro et al.^23^	To evaluate inflammatory and oxidative stress biomarkers in subjects exposed to silica	Cross-sectional	34 crystal craftsmen currently exposed, 35 formerly exposed, and 12 nonexposed	BMP2; chemokines CXCL16 and CCL5	Brazil
Braz et al.^24^	To evaluate plasma levels of biomarkers	Cross-sectional	57 subjects exposed to silica, 36 with silicosis, and 22 unexposed	CCL2, CCL3, CCL11, CCL24; TNF-α, sTNFR1, sTNFR2; eotaxin	Brazil
Braz et al.^25^	To evaluate plasma levels of inflammatory mediators in subjects exposed to silica	Cross-sectional	30 subjects exposed to silica, 24 unexposed	IL-6, IL-1b, IL-10, TNF-a, sTNFR or sTNFR2	Brazil
Ophir et al.^26^	To demonstrate the direct effect of ultrafine particles in the lungs of workers exposed to artificial stone dust	Cross-sectional	68 workers exposed and 48 nonexposed individuals	IL-6, IL-8; TNF-α	Israel
Anlar et al.^27^	To investigate the effects of occupational silica exposure on oxidative stress parameters	Cross-sectional	99 male Turkish ceramics workers and 81 unexposed male office workers	SOD; CAT; glutathione; GR; GPx; GSH; TBARS; IL-1α, IL-1β, IL-2, IL-4, IL-6, IL-10; TNF-α	Turkey
Ganesan et al.^28^	To investigate modulation by silica nanoparticles (SiNPs) and evaluate cytokine release profiles and immunoglobulin levels across different exposures to SiNPs	Cross-sectional	4 patients with silicosis and 4 unexposed healthy controls	IgM and IgG; IL-1b, IL-6, IL-10, IL-4 and IFN-γ	Belgium
Jiang et al.^29^	To examine plasma levels of TNF-α and MMP-9	Cross-sectional	30 unexposed healthy controls, 28 individuals exposed to silica but without silicosis, and 30 with silicosis	TNF-α; MMP-9	China
Miao et al.^30^	To assess change in proteomic profile during the early stages of silicosis	Cross-sectional	15 unexposed healthy controls, 15 individuals exposed to silica but without silicosis, and 15 with silicosis	TNFs, IFN-β precursor, IL-6, TNFR13BV, and IL-17F	China
Sun et al.^31^	To investigate the therapeutic effect of N-acetylcysteine combined with tetrandrine	Cross-sectional	196 patients with silicosis; 108 received routine treatment and 88 received tetrandrine combined with N-acetylcysteine	IL-6; TNF‑α	China
Tan et al.^32^	To investigate whether exposure to lipopolysaccharides (LPS) can exacerbate fibrosis	Cross-sectional	12 male workers exposed to silica	Cleaved caspase-3; IL-1β, IL-6, and TNF-α	China
Liu et al.^33^	To analyze the inflammatory response in workers with silicosis	Cross-sectional	12 workers with stage I silicosis, 17 with stage II silicosis, 30 with stage III silicosis, and 14 healthy controls	sRAGE; TNF-α, IL-1β, IL-6, TGF-β1, and LDL-ox	China
Zhang et al.^34^	To investigate levels of Clara cell 16-kDa protein and IL-12 in bronchoalveolar lavage fluid	Cross-sectional	79 patients with silicosis at various stages: 41 with stage I silicosis, 25 with stage II silicosis, and 13 with stage III silicosis	CC16; IL-12	China

AAT = alfa-1 antitrypsin; BMP2 = bone morphogenetic protein 2; CAT = catalase; CC16 = Clara cell protein 16; CCL = chemokine ligand; GPx = glutathione peroxidase; GR = glutathione reductase; GSH = glutathione; IL = interleukin; IL2R = IL-2 receptor; LDH = lactate dehydrogenase; LPS = lipopolysaccharide; MMP = matrix metalloproteinase; CRP = C-reactive protein; SiNPs = silica nanoparticles; SOD = superoxide dismutase; sRAGE = soluble receptor for advanced glycation end-products; sTNFR = soluble tumor necrosis factor (TNF) receptor; TBARS = thiobarbituric acid reactive substances; TGF = transforming growth factor ; IgM = immunoglobulin M; IgG = immunoglobulin G; IFN = interferon; LDL = low-density lipoprotein.

## DISCUSSION

This integrative review on inflammatory biomarkers in silicosis found few studies on the topic; most were from China (six studies), followed by Brazil (three studies), Turkey, Spain, Israel, and Belgium (one study each), for a total of 13 eligible studies published between 2012 and 2022.

In a prospective study carried out between January 2009 and December 2018 in337 silica-exposed workers with a history of at least 5 years of occupational exposure - 278 of them with silicosis - and 30 individuals in the control group, Blanco-Pérez et al.^22^ analyzed a range of biomarkers. The authors found that levels of IL-8, alpha 1-antitrypsin (AAT), ferritin, C-reactive protein (CRP), and lactate dehydrogenase (LDH) were higher in workers with silicosis than in those exposed to silica but without silicosis. Patients exposed to silica with an established diagnosis of silicosis had higher levels of IL-2R, IL-6, and IL-8 than healthy controls. Patients diagnosed with complicated silicosis had higher levels of IL-2R, IL-6, IL-8, AAT, ferritin, CRP, and LDH than those diagnosed with simple silicosis. IL-8, LDH, and AAT levels were associated with progression of silicosis, and IL-6, IL-8, LDH, AAT, ferritin, and CRP levels were associated with a fatal outcome.^22^

On analysis of biomarker concentrations in the three groups (silica-exposed, simple silicosis, and complicated silicosis), only IL-8 differed across groups. LDH, AAT, ferritin, and PCR differed significantly between the complicated silicosis, silica-exposed, and simple silicosis groups, but not between silica-exposed and simple silicosis groups. IL-6 differed between the complicated silicosis and simple silicosis groups, with higher serum levels in patients with or without silicosis than in unexposed healthy individuals.^22^

IL-6 is a multifunctional cytokine recognized as the main mediator of the acute phase response with anti-inflammatory effects, exerting control over IL-1 and TNF production. TNF has the role of inducing influx of inflammatory cells to the site of injury, promoting secretion of other cytokines and enhancing fibroblast proliferation and collagen deposition. Analysis of IL-8, a neutrophil chemotactic factor, suggested its potential as a biomarker for presence of silicosis and in predicting mortality. This was the first study to analyze the clinical utility of a variety of serum biomarkers in a broad cohort of individuals exposed to silica dust, with and without silicosis. The authors also distinguished patients with complicated silicosis from those with simple silicosis.^22^

In a study carried out by Scalia Carneiro et al.^23^ in Brazil, CXCL16 emerged as a potential biomarker particularly to distinguish between levels of radiological severity, exhibiting a dose-response gradient from individuals with silicosis to silica-exposed and unexposed individuals. This biomarker is produced in large quantities by alveolar macrophages in the bronchial epithelium, and expression of its receptor (CXCR6) is increased in the BALF and T cells of individuals with asthma and sarcoidosis.

Another study carried out in Brazil, by Braz et al.,^24^ evaluated plasma levels of several inflammatory biomarkers and found significantly elevated levels of CCL24 in workers with silicosis compared to the control group, suggesting that CCL24 plays a role in the pathogenesis of silicosis. However, it is important to note that some diseases, such as asthma, are associated with increased plasma levels of CCL24, which reinforces the view that this chemokine is not specific for silicosis but rather may function as an inflammatory marker. Furthermore, the study showed a positive correlation between soluble TNFR1 (sTNFR1) and sTNFR2 and the radiological severity and time of exposure to silica. sTNFR2 was associated with all radiological severity categories in silicosis. This study concluded that measurement of sTNFRs may be useful in detecting silica exposure.

Another study carried out by Braz et al.^25^ found that plasma levels of IL-6 were higher in individuals exposed to silica, with or without silicosis, than in the control group. There was a positive correlation between radiological severity and quality of life, while a negative correlation was observed between radiological severity and lung function. The authors also found a negative correlation between plasma levels of sTNFR1 and lung functional capacity. IL-10 correlated negatively with quality of life and positively with lung functional capacity and the 6-minute walk test. No differences were found in plasma IL-1β, IL-10, TNF-α, sTNFR1, or sTNFR2 levels between the two studied groups.

A study carried out in Israel by Ophir et al.^26^ evaluated ultrafine silica particles in sputum supernatant from silica-exposed workers. They tested for correlations with the inflammatory biomarkers IL-6, IL-8, and TNF-α. The study found an association between ultrafine particles in workers’ lungs and a decrease in total lung capacity, deterioration on lung computed tomography, and elevation of the cytokines of interest.

Anlar et al.^27^ assessed IL-1α, IL-1β, IL-2, IL-4, IL-6, IL-10, and TNF-α levels in Turkish ceramic workers. In this study of 99 workers, almost 50% were diagnosed with silicosis; of these, 84% had category 1 silicosis. The workers had significantly higher levels of IL-1α, IL-1β, IL-2, IL-4, IL-6, IL-10, and TNF-α. Workers with a longer duration of exposure (> 16 years) had significantly higher IL-6 levels than other workers. Furthermore, workers over 42 years of age had significantly higher IL-1α levels than younger workers. This may be attributable to cumulative inflammatory effects from dust exposure, indicative of an inflammatory response.

A Belgian study carried out by Ganesan et al.^28^ assessed the responsiveness of peripheral blood mononuclear cells (PBMCs]) to silica nanoparticles (SiNPs) in four patients with silicosis and four nonexposed healthy controls. This study evaluated the cytokines IL-1β, IL-6 and IFN-γ, and found them to be upregulated in PBMCs when stimulated by SiNPs of patients compared to controls. Without stimulation, cytokine levels did not differ significantly between patients and controls, except for IFN-γ and IL-17, which, although not significantly so, were higher in patients, and IL-4 and IL-10, which were higher in controls. M1 macrophages are dominant in the early stage of inflammation and accompanied by high expression of inflammatory cytokines, such as TNF-α, IL-1β, and IL-6, while M2 macrophages appear dominant in the late fibrosis stage, with attendant overexpression of the anti-inflammatory cytokine IL-10. It bears stressing that cytokines such as TNF-α and IL-1β are also secreted abundantly by macrophages in response to silica and, therefore, may also participate in the perpetuation of inflammation induced by SiNP.

One study conducted in China by Jiang et al.^29^ evaluated plasma levels of TNF-α and matrix metalloproteinase-9 (MMP-9) in exposed groups with and without silicosis as well as in healthy controls and found increased levels in both exposed groups (with and without silicosis), although they were highest in patients with silicosis. TNF-α is an important cytokine initiator of inflammatory responses, and several studies show its production is increased in mononuclear cells from patients with silicosis. MMP-9 regulates cell differentiation and proliferation, and MMP-9 levels are upregulated by TNF-α. Thus, both cytokines may be implicated in the development of silicosis.^29^

In 2016, Miao et al.^30^ evaluated differences in proteomic profile between healthy individuals and dust-exposed workers with and without silicosis. They found significant changes in serine proteases, glycoproteins, and proto-oncogenes that may be associated with upregulation of structural constituents of the extracellular matrix, immune response, and fibroblast proliferation. Upregulation of cytokines including TNFs, IFN-β precursor, IL-6, atypical chemokine receptor 2, TNFR13BV, and mutant IL-17F may be implicated in the exacerbated, persistent immune response and fibrosis that occur during the development of silicosis.

Cytokines are a broad group of small proteins or peptides that are released from immune cells and are involved in cell signaling and regulation of interactions in the development and differentiation of the immune response. The study found that numerous cytokines, including ILs, IFNs, chemokines and TNFs, were more abundant in dust-exposed patients with and without silicosis than in the unexposed group. Miao et al.^30^ also found that TNF ligand superfamily 4 isoform X1 (TNFSF4), IFN-β precursor, IL-6, atypical chemokine 2 receptor, and mutant IL-17F were all upregulated in exposed patients, both with and without silicosis.

Sun et al.^31^ evaluated the clinical efficacy of combined N-acetylcysteine and tetrandrine tablets in the treatment of silicosis and its effect on serum levels of IL-6 and TNF-α in patients with silicosis. Patients were divided into two groups according to treatment method. A control group of 108 patients received routine treatment, including anti-inflammatory therapy, while the 88 patients in the observation group were treated with a combination of tetrandrine and N-acetylcysteine.

There was no significant difference in treatment efficacy between the two groups. There was no significant difference in serum levels of IL-6 and TNF-α between the two groups before treatment. After treatment, IL-6 and TNF-α levels in the two groups decreased significantly, and levels of both cytokines in the observation group after treatment were significantly lower than those in the control group. The authors concluded that the combination of tetrandrine and acetylcysteine has an improved therapeutic effect on silicosis and can mitigate the severity of the inflammatory reaction in patients with this disease, and that levels of IL-6 and TNF-α in peripheral blood may be valuable to guide clinical treatment of silicosis.^31^

Tan et al.^32^ investigated the potential role of exposure to lipopolysaccharide (LPS) in silicosis. They collected alveolar macrophages from 12 male workers exposed to silica and incubated these cells in the presence and absence of LPS for 24 hours. The authors found that levels of cleaved caspase-3 and of the pro-inflammatory cytokines IL-1β, IL-6, and TNF-α were increased in these macrophages after LPS exposure. LPS are characteristic components of the cell wall of Gram-negative bacteria. There is growing evidence that LPS may worsen a wide range of diseases, such as Alzheimer’s disease and Parkinson’s disease, damage the reproductive system, and cause liver toxicity by promoting apoptosis and inflammation. Additionally, a previous study detected LPS in BALF from patients with silicosis. Thus, there is an urgent need to elucidate whether LPS can stimulate production of inflammatory cytokines. The authors’ findings suggest that LPS aggravates the inflammatory response in patients with silicosis.

Liu et al.,^33^ in a study conducted in China, analyzed secretion of the soluble receptor for advanced glycation end products (sRAGE), TNF-α, IL-1β, IL-6, and TGF-β1 in the serum of individuals with silicosis (n = 59) and healthy controls (n = 14). TNF-α, IL-1β, IL-6, and TGF-β1 levels were significantly increased in the silicosis group compared to the healthy control group. Additionally, sRAGE levels correlated negatively with TNF-α, IL-6, and IL-1β.

No correlation was found between sRAGE and TGF-β1 and lung function. Workers who had longer occupational exposure to silica had higher levels of sRAGE.^33^

Zhang et al.^34^ evaluated 41 patients with stage I silicosis, 25 patients with stage II silicosis, and 13 patients with stage III silicosis, with age ranging from 33 to 67 (mean, 48.6±3.9) years and exposure time from 5 to 27 (mean, 12.7±6.5) years, as well as a comparator group of 20 workers with no pneumoconiosis despite similar duration of exposure and type of work. All underwent bronchoalveolar lavage. After comparison, there was no significant difference in age or exposure time between the control group and the silicosis group. IL-12 levels in BALF in the stage I, II, and III silicosis groups were higher than those in the control group, while IL-12 levels in stage II and III silicosis were higher than those in stage I patients.

The likely reason proposed by the authors is that silica dust exposure is a stimulus that abnormally activates macrophages, resulting in increased secretion of IL-12. Levels of Clara cell protein 16 (CC16) and IL-12 in the BALF of silicotic patients in the < 10-year group were higher than those in the 10-to-20-year group and the > 20-year group. The authors’ analysis showed a positive correlation between CC16 and IL-12 levels and exposure time in the silicosis group. In the control group, CC16 and IL-12 levels did not show statistically significant differences.^34^

## FINAL CONSIDERATIONS

The concentration of studies in China may be associated with some limitations, although China is the country with the most mining companies in the world. Due to the dearth of studies, it is possible that the evidence compiled in this integrative review is not robust enough for definitive conclusions to be drawn. Furthermore, the lack of geographic diversity in the study sample may limit the generalization of findings to other populations and settings. Nevertheless, this integrative review was able to identify how cytokines - the inflammatory biomarkers which were the object of this study - are associated with silica exposure and silicosis. This may help inform future research and guide the development of new therapies and interventions for this disease.

The results of this integrative review also indicate a need for additional research in different countries and regions to better understand variations in inflammatory biomarkers and their potential effects on silicosis, consequently leading to better diagnosis, earlier diagnosis, and early treatment of silicosis worldwide. Longitudinal follow-up of patients will be of the utmost importance for identifying key inflammatory markers of exposure and disease severity, as well as to identify whether those with increased levels of these biomarkers are more likely to develop silicosis over the years.
